# Hydrogen Storage Performance of γ-Graphdiyne Doped Li Based on First Principles for Micro/Nano

**DOI:** 10.3390/mi13040547

**Published:** 2022-03-30

**Authors:** Wenchao Tian, Zhao Li, Chunmin Cheng, Wenhua Li, Zhiqiang Chen, Fei Xin

**Affiliations:** School of Electro-Mechanical Engineering, Xidian University, Xi’an 710000, China; zhaoli960628@163.com (Z.L.); cmcheng@whu.edu.cn (C.C.); whli@stu.xidian.edu.cn (W.L.); zqchen@xidian.edu.cn (Z.C.); fxin@xidian.edu.cn (F.X.)

**Keywords:** γ-GDY, first-principles, dope, vacancy defect, hydrogen storage property

## Abstract

The rapid development of micro/nano systems promotes the progress of micro energy storage devices. As one of the most significant representatives of micro energy storage devices, micro hydrogen fuel cells were initially studied by many laboratories and companies. However, hydrogen storage problems have restricted its further commercialization. The γ-graphdiyne (γ-GDY) has broad application prospects in the fields of energy storage and gas adsorption due to its unique structure with rigid nano-network and numerous uniform pores. However, the existence of various defects in γ-GDY caused varying degrees of influence on gas adsorption performance. In this study, Lithium (Li) was added into the intrinsic γ-GDY and vacancy defect γ-GDY (γ-VGDY) to obtain the Li-GDY and Li-VGDY, respectively. The first-principles calculation method was applied and the hydrogen storage performances of them were analysed. The results indicated that the best adsorption point of intrinsic γ-GDY is H2 point, which located at the centre of a large triangular hole of an acetylene chain. With large capacity hydrogen storage, doping Li atom could improve the hydrogen adsorption property of intrinsic γ-GDY; meanwhile, vacancy defect inspires the hydrogen storage performance further of Li-VGDY. The mass hydrogen storage density for Li_2_H_56_-GDY and *Li*_2_H_56_-VGDY model were 13.02% and 14.66%, respectively. Moreover, the Li_2_H_56_-GDY and *Li*_2_H_56_-VGDY model had same volumetric storage density, with values that could achieve 5.22 × 10^4^ kg/m^3^.

## 1. Introduction

Micro energy storage devices are the power core of micro/nano systems, which have been widely used in the field of microelectromechanical systems, micro/nano robots, smart portable/wearable systems and micro-implantable medical sensors, etc. The development of micro and nano technology drives the developing trend of micro/nano systems towards miniaturization, systematic and intelligence, etc. Thus, higher requirements have been proposed for its energy storage components, such as micro volume, high power and long reliability etc. [1,2,3,4].

Micro batteries, such as Li+ [5] micro batteries, Na+ [6] micro batteries and micro fuel cells [7], are common micro energy storage devices in the micro/nano system. Among them, micro fuel cells have been regarded as one of the key technologies in the field of energy due to their excellent advantages with high energy conversion efficiency, large output power and non-pollution, etc. In addition, as an ideal secondary energy, hydrogen energy has recently been considered as a green and strategic energy by the international community [8,9,10]. Therefore, the research and development of hydrogen fuel cells has been widely accepted by many scholars and laboratories. Meanwhile, many companies have conducted research for the commercialization of micro fuel cells actively [11]. However, in order to meet the requirements of portability and high energy density for the hydrogen micro fuel cell, it is critical to solve the problem of hydrogen storage [12,13,14].

Hydrogen storage methods mainly include high-pressure gaseous hydrogen storage, liquid hydrogen storage and solid hydrogen storage. Solid hydrogen storage in particular can effectively overcome the shortage of gaseous and liquid hydrogen storage with convenient transportation, high storage density and safety coefficient, etc. Carbon-based hydrogen storage materials, such as carbon nanotubes [15], activated carbon [16] and graphene [17] etc., were considered as promising solid hydrogen storage materials due to its outstanding hydrogen adsorption and release properties. Yang et al. [18] studied the hydrogen storage property of a sandwich structure composed of a carbon nitride and two graphene sheets based on the first principles. The results indicated that hydrogen could be blocked by the graphene completely and stored in this sandwich structure with higher density. Liu et al. [19] studied the hydrogen adsorption performance of Li decorated C68-GY. The results indicated that the hydrogen storage density is 8.04 wt% with adsorption energy of −0.227. Moreover, hydrogen binds with Li/C through polarization and H–H interaction among molecules. Cui et al. [20] and Liu et al. [21] studied hydrogen storage property of Ti decorated double vacancy graphene and porous graphene, respectively. Calculation results shown that the hydrogen adsorption capacity of decreased 33% after doped with Ti, in contrast, Ti-porous graphene system possessed excellent hydrogen storage property.

Graphdiyne (GDY), as a new type of all-carbon molecule, was synthesized through an in situ chemical method for the first time on a copper surface according to the research of Li et al. [22]. It has a unique 1,3-diacetylene bond [23], which can conjugate with the benzene ring to form a two-dimensional planar network structure. Meanwhile, the large triangular rings in GDY formed by alkyne bond and benzene ring improved the pore size to 0.25 nm [24]. In addition, the GDY has many excellent properties, such as, rich carbon chemical bonds, large conjugate system and better ion shuttling etc., which can maintain the long reliability of device [25]. Due to these advantages, the GDY has huge potential for its application in the field of hydrogen storage. Zhang et al. [26] investigated the intercalation and diffusion of hydrogen in the single layer and bulk GDY. The results for the first time revealed that the GDY possessed excellent hydrogen storage performance. Panigrahi et al. [27] studied the hydrogen storage property of GDY doped with light metal (Li, Na, K, Ca, Sc and Ti). Computation results showed that the Li-GDY possessed the highest hydrogen storage capacity, which reached 6.5 wt%.

Many studies of hydrogen storage equipment have focused on carbon materials, especially in the *GDY* materials. In this study, the hydrogen absorption properties of *GDY* modified structures doped with Li or vacancy defect were investigated. The calculation results were essential to understanding and analysing the adsorption mechanism and broadening the practical applications in the field of hydrogen storage.

## 2. Simulation Methods and Computation Models

### 2.1. Simulation Methods

In this study, the first-principles calculation method based on density functional theory was adopted, and CASTEP program package in Materials Studio software was used. The electron exchange correlation potential selected the Perdew–Burke–Ernzerhof (PBE) functional of generalized gradient approximation (GGA) [28,29]. The X and Y directions were superposed and the Z direction was erected to the graphene plane, respectively, to obtain calculation results. The vacuum layer was 20 Å. In order to acquire stable total system energy, considering the factors of calculation accuracy and efficiency, the plane wave truncation energy with 600 eV and the Brillouin zone K point of Monkhorst-Pack with 6 × 6 × 1 were selected, respectively. The above simulation parameters can ensure that the total energy error of the system convergence was not more than 2 × 10^−5^ eV/atom.

### 2.2. Computation Models

**Figure 1:**
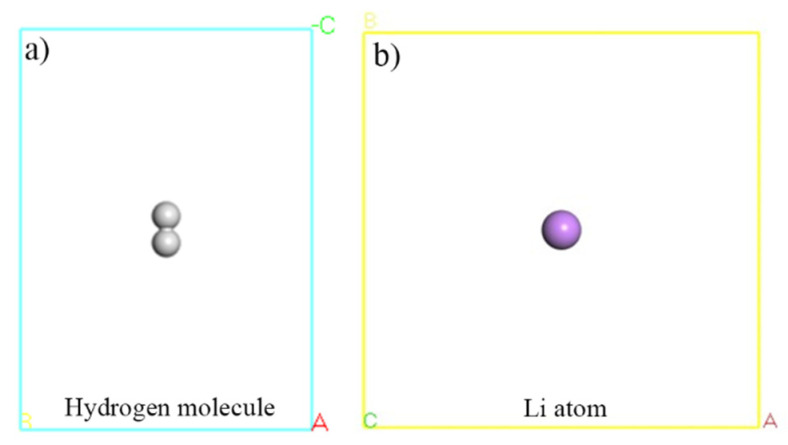
Computation models of hydrogen molecule and Li atom.

Figure 1a,b show the computation models of hydrogen molecule and Li atom, respectively. The optimized structures of hydrogen molecule and Li atom were obtained through the CASTEP of Materials studio. The steady-state energy of hydrogen molecule ($E_{H_{2}}$) was −31.67 eV, and the bond length of hydrogen-hydrogen was 0.746 Å. The steady-state energy of Li atom ($E_{Li}$) was −188.20 eV.

**Figure 2:**
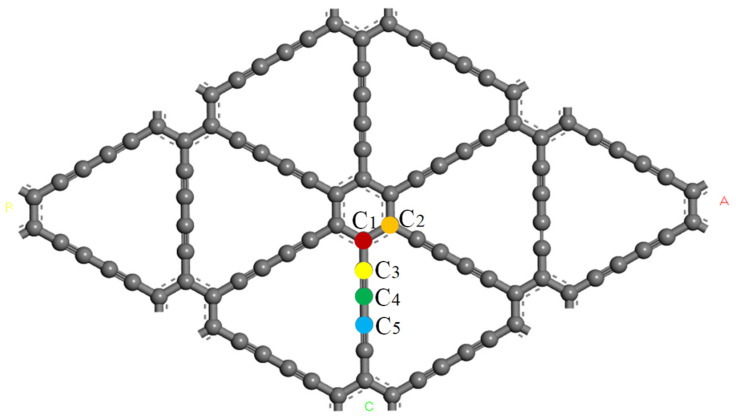
Supercell structure of intrinsic γ-GDY models.

Figure 2 shows the initial simulation model of intrinsic γ-GDY, which is a 2 × 2 supercell, and the steady-state energy is −11,111.919 eV. In this figure, there are four different carbon–carbon (C-C) bonds, and the bond length of aromatic bond (C_1_-C_2_), C–C single bond (C_1_-C_3_), C–C triple bond (C_3_-C_4_), and C–C single bond (C_4_-C_5_) are 1.430 Å, 1.392 Å, 1.232 Å and 1.334 Å, respectively. Several related research reported the C–C bond length of γ-GDY and their results are listed in Table 1, which proved that the intrinsic γ-GDY in this study is reasonable and could be used to simulate the hydrogen adsorption process.

**Figure 3:**
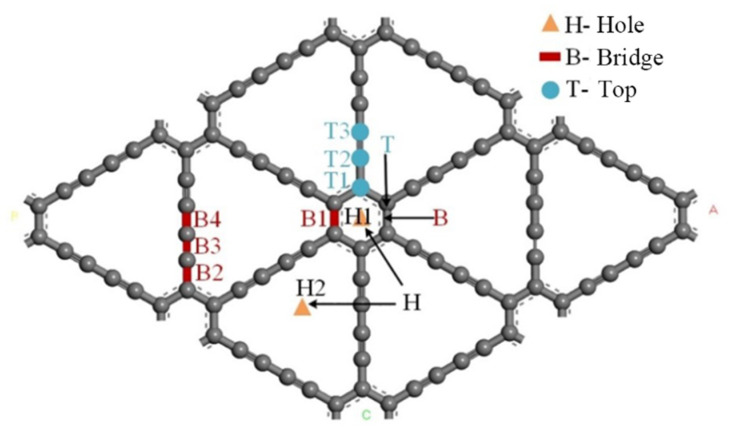
Schematic diagram of the nine highly symmetric adsorption points of intrinsic γ-GDY models.

Due to the particularity of the acetylene bond in the intrinsic γ-GDY structure and the similarities and differences of the C–C bond length, nine highly symmetric adsorption points were selected during the hydrogen adsorption process. Figure 3 shows the schematic diagram of nine highly symmetric adsorption points of the γ-GDY models. According to the difference location of points, three groups are divided as presented in Figure 3. Two points located at the centre of the pore were defined as hole (H). Bridge (B) referred four points in the middle of the C–C bond. Additionally, top (T) showed three points at the directly above of the carbon (C) atom. The supercell structure of the γ-GDY was the critical factor for all points selection. H1 and H2 points were located in the centre of the benzene ring and the large triangular hole composed of the acetylene chain, respectively, and the selection of them based on all pores structure of the supercell structure. The types of all C–C bonds in the supercell structure were the crucial selection factors of B points. The C-C bond of the benzene ring referred to B1 points, the C–C single bond and triple bonds of the ethyne chain were defined as B2 and B3 points, respectively, and B4 points showed the C–C single bond which connected two C–C triple bonds. According to the hybridization mode of different C atoms in the supercell structure, the C atom on the benzene ring was selected as T1 point, and the two C atoms on the ethyne bond were selected as T2 and T3 points, respectively.

**Figure 4:**
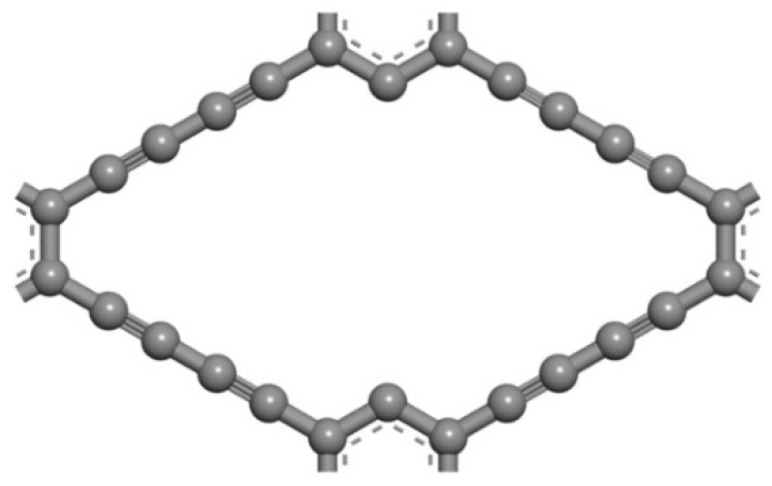
Schematic diagram of the γ-VGDY models.

Vacancy defect occurs in carbon materials inevitably. As shown in Figure 2 and Figure 3, the intrinsic γ–GDY is divided into two symmetrical parts by the ethyne chain. The missing ethyne chain forms a new structure, known as vacancy defect γ-GDY (γ-VGDY), composed of four identical ethyne chains and a benzene ring. In order to analyse the effect of vacancy defect on the hydrogen storage performance, the γ-VGDY model is also established by the Materials studio software, as shown in Figure 4.

## 3. Results and Discussion

### 3.1. Hydrogen Adsorption Property of Intrinsic γ-GDY

**Figure 5:**
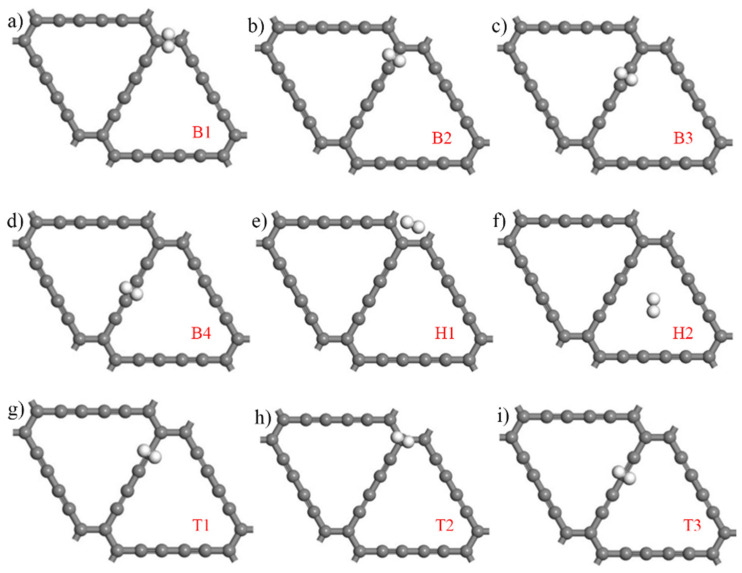
Hydrogen adsorption models of intrinsic γ-GDY at nine highly symmetric adsorption points. (Hydrogen molecules are expressed by white balls, (**a**–**i**) represent the hydrogen molecule adsorption points B1, B2, B3, B4, H1, H2, T1, T2 and T3, respectively).

In order to investigate the hydrogen adsorption property of intrinsic γ-GDY, the hydrogen adsorption models of nine highly symmetric adsorption points are shown in Figure 5. The concentration of hydrogen molecule and initial adsorption height were set as 1 and 3 Å severally to obtain optimal hydrogen storage property. The hydrogen adsorption energy of intrinsic γ-GDY at nine highly symmetric adsorption points was also calculated, which can be expressed in Equation (1):

(1) $$\;E_{ads}=E_{GDY+H_{2}}-E_{GDY}-E_{H_{2}}\;$$

Here, $E_{GDY+H_{2\;}}$is the total energy of intrinsic γ-GDY after adsorbing one hydrogen molecule, the $E_{GDY}$ and $E_{H_{2}}$ are the energy of intrinsic γ-GDY and single hydrogen molecule, respectively. If the adsorption energy is negative, it means that the system releases heat during the adsorption process, and the total energy of the system is reduced and the adsorption process more easily occurs. Thus, the higher the absolute value is, the easier the adsorption becomes. In contrast, when the adsorption energy is positive, this process needs to absorb heat, the higher absolute value shows that the adsorption of hydrogen is difficult. Table 2 shows the adsorption height and energy of nine points. As shown in this table, the H2 point possesses the largest absolute value of adsorption energy and lowest adsorption height for hydrogen, thus the H2 point is seen as the best adsorption point for intrinsic γ-GDY.

### 3.2. Li Adsorption Property of Intrinsic γ-GDY and γ-VGDY

**Figure 6:**
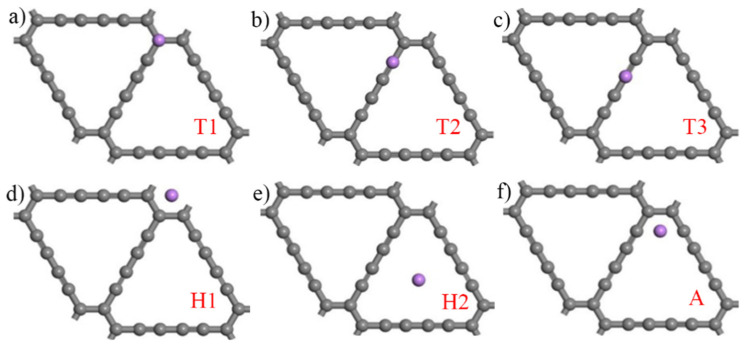
Six initial Li-GDY models (Purple ball is Li atom, (**a**–**f**) represent the Li atom adsorption points T1, T2, T3, H1, H2 and A, respectively).

The H2 point shows the best performance of hydrogen adsorption in nine adsorption points, however, the lower adsorption energy indicates that the hydrogen storage property of intrinsic γ-GDY is not ideal. Thus, it is necessary to study the hydrogen storage property of intrinsic γ-GDY doping with Li (Li-GDY). Figure 6 shows the initial Li atom adsorption models of intrinsic γ-GDY, which are named as the initial Li-GDY models. According to Table 2, the difference of adsorption energy values for B and T points are limited, thus the T and H points are selected to establish the adsorption models. Moreover, the Angle point (A) was introduced to enrich the diversity of simulation models. The adsorption energy of intrinsic γ-GDY to Li atom can be expressed in Equation (2):

(2) $$\;E_{ads}=E_{GDY+Li}-E_{GDY}-E_{Li}\;$$

Here, $E_{GDY+Li}\;$is the total energy of intrinsic γ-GDY after adsorbing one Li atom, the $E_{GDY}$ and$\;E_{Li}$ are the energy of intrinsic γ-GDY and single Li atom, respectively. The adsorption height and energy of intrinsic γ-GDY after doping Li atom is shown in Table 3. In this table, the adsorption energy of T1, T2, T3 and H1 points to Li atom are −1.991 eV, −1.983 eV, −2.021 eV and −1.979 eV, respectively. Meanwhile, the adsorption energy of H2 and A points to Li atom are −2.657 eV and −2.641 eV, respectively. The results indicate that the H2 and A points of intrinsic γ-GDY are the best dope points when doping a single Li atom. In the following simulation, the H2 point of intrinsic γ-GDY is selected as the dope point for the Li atom and the adsorption height is 0.084 Å.

**Figure 7:**
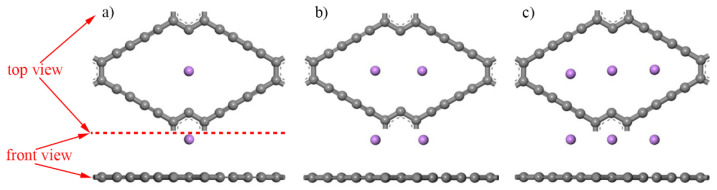
Three initial Li-VGDY models. (Purple ball is Li atom, (**a**–**c**) represent the Li atoms concentration 1, 2 and 3, respectively).

The analogous rhombic ring composed of four ethyne chains in γ-VGDY was the optimal dope area. Thus, the doping concentration of Li atoms was considered as the significant factor. Figure 7 shows the initial γ-VGDY models when doping different concentration Li atoms (Li-VGDY), which are named as the initial Li-VGDY models. The average adsorption energy of γ-VGDY to Li atoms can be expressed in Equation (3):

(3) $$\bar{E_{ads}}=\left(E_{\left(x\right)Li+VGDY}-E_{VGDY}-xE_{Li}\right)/x\;$$

Here, $E_{\left(x\right)Li+VGDY}$ is the total energy of γ-VGDY after doping *x* Li atoms, the $E_{VGDY}$ and$\;E_{Li}$ are the energy of γ-VGDY and a single Li atom, respectively, *x* is the number of Li atoms. Table 4 shows the adsorption height and average adsorption energy of γ-VGDY after doping Li atoms. In this table, the average adsorption energy of γ-VGDY after doping 1, 2 and 3 Li atoms are −4.437 eV, −3.720 eV and −3.130 eV, respectively. Li-VGDY model possesses the maximal average adsorption energy, thus it is selected in the following simulation and the adsorption height of Li atom is 0.396 Å. In addition, compared with γ-GDY, the γ-VGDY expresses better doping ability of Li atoms.

### 3.3. Hydrogen Adsorption Property of Li-GDY and Li-VGDY

**Figure 8:**
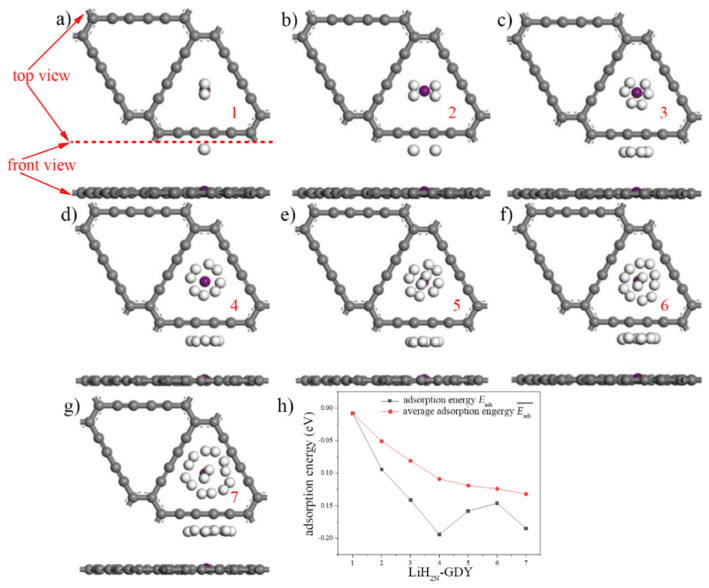
Seven LiH_2N_-GDY models and adsorption energy. (Purple ball is Li atom, white balls are hydrogen molecules, (**a**–**h**) represent the LiH_2N_-GDY models of hydrogen concentrations with 1, 2, 3, 4, 5, 6 and 7, respectively, (**h**) shows the hydrogen adsorption energy and average adsorption energy of Li-GDY).

Because of the unacceptable hydrogen storage performance of intrinsic γ-GDY, the structure and energy of Li-GDY computation models were optimized. The adsorption characteristic parameters of LiH_2N_-GDY system under the different density of hydrogen molecules were studied. Figure 8a–g show the hydrogen adsorption models of Li-GDY under different hydrogen concentrations (LiH_2N_-GDY), which are named as the LiH_2N_-GDY models. Graphene materials are permeable to hydrogen, even though it is defect free [35]. However, if hydrogen could be adsorbed on the γ-GDY by chemical bonding, it will be difficult to achieve the function of hydrogen permeation. Moreover, in order to determine the stability of hydrogen molecule adsorption on the Li-GDY surface, the adsorption energy of hydrogen ($E_{ads}$) was also calculated, which can be expressed in Equation (4) [19,36]:

(4) $$E_{ads}=E_{GDY+\left(x\right)Li+\left(N\right)H_{2}}-E_{GDY+\left(x\right)Li+\left(N-1\right)H_{2}}-E_{H_{2}}$$

The average adsorption energy of hydrogen ($\bar{E_{ads}}$) can be described in Equation (5):

(5) $$\bar{E_{ads}}=\left(E_{GDY+\left(x\right)Li+\left(N\right)H_{2}}-E_{GDY+\left(x\right)Li}-E_{\left(N\right)H_{2}}\right)/N$$

Here, $E_{GDY+\left(x\right)Li+\left(N\right)H_{2}\;}$and $E_{GDY+\left(x\right)Li+\left(N-1\right)H_{2}}\;$are the total energy of intrinsic γ-GDY doped *x* Li after adsorbing *N* and *N* − 1 hydrogen molecules, respectively. $E_{GDY+\left(x\right)Li}$ is the total supercell energy of intrinsic γ-GDY doped *x* Li. $E_{H_{2}}$ and $E_{\left(N\right)H_{2}}$ are the energy of single and *N* hydrogen molecules, respectively, and *N* is the number of hydrogen molecules.

Table 5 shows the hydrogen adsorption energy and average adsorption energy of Li-GDY. As shown in this table, when the concentration of hydrogen molecules exceeds three, the absolute value of average adsorption energy of Li-GDY surpasses the absolute value of adsorption energy of intrinsic γ-GDY in H2 point. This phenomenon indicates that Li-GDY shows better hydrogen storage property compared to the intrinsic γ-GDY when in large capacity hydrogen storage. Thus, the hydrogen storage property of intrinsic γ-GDY could be improved by the doping Li atom. When the hydrogen molecular concentration is four, the adsorption energy reaches the peak, and the value is −0.194 eV. Moreover, the hydrogen average adsorption energy of Li-GDY increases obviously with the rise in molecular concentration. When the number of hydrogen molecules are seven, the best average adsorption energy occurs, which can achieve −0.132 eV.

**Figure 9:**
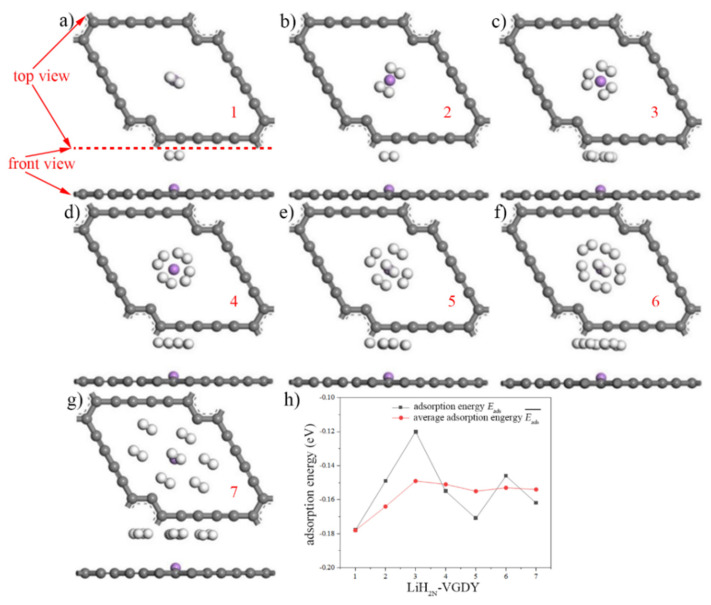
Seven LiH_2N_-VGDY models and adsorption energy. (Purple ball is Li atom, white ball is hydrogen molecule, (**a**–**h**) represent the LiH_2N_-VGDY models of hydrogen concentrations with 1, 2, 3, 4, 5, 6 and 7, respectively, (**h**) shows the hydrogen adsorption energy and average adsorption energy of Li-VGDY).

In order to analyse the hydrogen storage property of Li-VGDY, the seven hydrogen adsorption models of Li-VGDY under different hydrogen concentrations (LiH_2N_-VGDY) are shown in Figure 9a–g. Meanwhile, the adsorption energy of hydrogen ($E_{ads}$) and average adsorption energy were calculated to determine the stability of hydrogen molecule adsorption on the Li-VGDY surface, which can be expressed by Equations (6) and (7):

(6) $$E_{ads}=E_{VGDY+\left(x\right)Li+\left(N\right)H_{2}}-E_{VGDY+\left(x\right)Li+\left(N-1\right)H_{2}}-E_{H_{2}}$$

(7) $$\bar{E_{ads}}=\left(E_{VGDY+\left(x\right)Li+\left(N\right)H_{2}}-E_{VGDY+\left(x\right)Li}-E_{\left(N\right)H_{2}}\right)/N$$

Here, $E_{VGDY+\left(x\right)Li+\left(N\right)H_{2}}\;$and $E_{VGDY+\left(x\right)Li+\left(N-1\right)H_{2}}\;$are the total energy of γ-VGDY doped *x* Li after adsorbing *N* and *N* − 1 hydrogen molecules, respectively. $E_{VGDY+\left(x\right)Li}$ is the total supercell energy of γ-VGDY doped *x* Li. $E_{H_{2}}$ and $E_{\left(N\right)H_{2}}$ are the energy of single and *N* hydrogen molecules, respectively, and *N* is the number of hydrogen molecules.

The hydrogen adsorption energy and average adsorption energy of Li-VGDY are shown in Table 6. In this table, different from the Li-GDY, the hydrogen adsorption energy of the Li-VGDY system shows fluctuation between −0.149 and 0.029 eV. In addition, the absolute value of average adsorption energy of Li-VGDY decreases with the increase in hydrogen molecular concentration. Nonetheless, the Li-VGDY possesses better average adsorption energy than Li-GDY. Meanwhile, the average adsorption energy of Li-VGDY reaches a steady state when the number of hydrogen molecules are four, which is −0.151 eV. The results indicate that the Li-VGDY has excellent stability in large capacity hydrogen storage.

### 3.4. The Capacity of Hydrogen Storage

High-efficiency hydrogen storage materials need to satisfy two significant conditions of larger storage capacity and higher bonding strength in the practical applications. The mass and volumetric hydrogen storage density were proposed by the US Department of Energy to measure the performance of hydrogen storage materials.

The mass hydrogen storage density ((η(wt%)) can be expressed in Equation (8):

(8) $$\eta \left(wt\%\right)=\frac{2Ar_{\left(H\right)}n\left(H_{2}\right)}{Ar_{\left(n\right)}\cdot n\left(c\right)+Ar_{\left(Li\right)}\cdot n\left(Li\right)+2Ar_{\left(H\right)}\cdot n\left(H_{2}\right)}\cdot 100\%$$

The volumetric hydrogen storage density (ρ(V)) can be described in Equation (9):

(9) $$\rho \left(V\right)=\frac{2Ar_{\left(H\right)}n\left(H_{2}\right)/N_{A}}{a\cdot b\cdot c}\cdot 100\%\;$$

Here, the *Ar*(*Li*), *Ar*(*H*) and *Ar*(*C*) are the relative atomic mass of Li, hydrogen and C atoms, respectively. *Ar*(*Li*) = 7, *Ar*(*H*) = 1 and *Ar*(*C*) = 12. The *n*(*Li*), *n*(*H*_2_) and *n*(*C*) are the number of Li atoms, hydrogen molecules and C atoms, respectively. *N*_A_ is the Avogadro constant, and the value is 6.022 × 10^23^. The *a*, *b* and *c* represented the overall length, width and height of the model, respectively.

**Figure 10:**
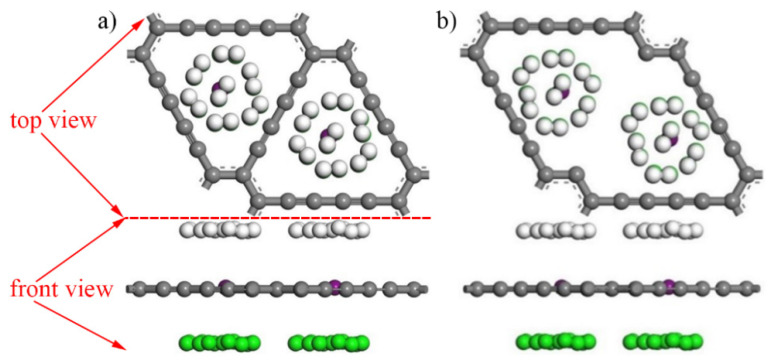
Maximum hydrogen storage capacity model of intrinsic γ-GDY and γ-VGDY. (**a**) Li_2_H_56_-GDY model, (**b**) Li_2_H_56_-VGDY model. (Hydrogen molecules on top and bottom surface are represented by white and green balls, the purple balls represent Li atoms).

According to the analysis of Table 5, when the concentration of hydrogen molecules is N = 7, the LiH_2N_-GDY system achieves the best performance of hydrogen storage. Figure 10 a shows the maximum hydrogen storage capacity model of intrinsic γ-GDY (Li_2_H_56_-GDY model). In this figure, two Li atoms are doped to the intrinsic γ-GDY, the top and bottom surface of Li-GDY adsorb seven hydrogen molecules, respectively, and the hydrogen molecules on top and bottom surface are represented by white and green balls, the purple balls represent Li atoms. The structure and energy of the model were optimized, and the adsorption energy of this system was −0.226 eV in steady state, which could meet the requirement of hydrogen storage adsorption energy. The mass and volumetric hydrogen storage densities of Li_2_H_56_-GDY system were 13.02% and 5.22 × 10^4^ kg/m^3^, and these values of intrinsic γ-GDY were 7.69% and 2.24 × 10^4^ kg/m^3^, respectively.

As shown in Table 6, when the number of hydrogen molecules is one, the performance of hydrogen storage of LiH_2N_-VGDY reaches its peak. In order to ensure the consistency of the simulation, here, the number of hydrogen molecules also selected seven. Figure 10b shows the maximum hydrogen storage capacity model of γ-VGDY (Li_2_H_56_-VGDY model). Computational results indicated that the adsorption energy of this system was −0.209 eV when in a steady state, which also could satisfy the requirement of hydrogen storage adsorption energy. Meanwhile, the mass hydrogen storage density of Li_2_H_56_-VGDY system was 14.66%, and the volumetric storage density was 5.22 × 10^4^ kg/m^3^, a value the same as the Li_2_H_56_-GDY system.

## 4. Conclusions

(1) The H2 point of intrinsic γ-GDY shows the best hydrogen adsorption property in nine highly symmetric adsorption points. However, the hydrogen adsorption performance of intrinsic γ-GDY is not ideal.
(2) Li atoms could be adsorbed by intrinsic γ-GDY and γ-VGDY stably.
(3) Doping Li atoms could enhance the hydrogen storage property of intrinsic γ-GDY when in large capacity hydrogen storage.
(4) Vacancy defect has a positive influence on hydrogen storage performance; Li-VGDY possesses better hydrogen storage performance than Li-GDY.

## Figures and Tables

**Table 1:** Related research of the C-C bond length of intrinsic γ-GDY.

References	C-C Single Bond (Å)	C-C Triple Bond (Å)	Aromatic Bond (Å)
Cranford [30]	1.460~1.480	1.180~1.190	1.480~1.500
Bai [31]	1.341~1.400	1.239	1.440
Mirnezhad [32]	1.404	1.219	1.423
Peng [33]	1.407	1.223	1.426
Pei [34]	1.340~1.400	1.230	1.430

**Table 2:** Hydrogen adsorption height and adsorption energy of nine points.

Points	B1	B2	B3	B4	H1	H2	T1	T2	T3
Adsorption height (Å)	3.0018	3.0019	3.1911	3.1528	2.9425	2.8159	3.1024	3.1760	3.1748
Adsorption energy (eV)	−0.0215	−0.0216	−0.0315	−0.0277	−0.0326	−0.0786	−0.0289	−0.0340	−0.0281

**Table 3:** Adsorption height and adsorption energy of six points for Li-GDY.

Points	T1	T2	T3	H1	H2	A
Adsorption height (Å)	1.930	1.931	1.906	1.878	0.084	0.232
Adsorption energy (eV)	−1.991	−1.983	−2.021	−1.979	−2.657	−2.641

**Table 4:** Adsorption height and adsorption energy of *Li*_(*x*)_-VGDY.

Concentration	1	2	3
Adsorption height (Å)	0.396	0.402	1.253
Average adsorption energy (eV)	−4.437	−3.720	−3.310

**Table 5:** Hydrogen adsorption energy and average adsorption energy of Li-GDY.

N (H_2_)	1	2	3	4	5	6	7
$E_{ads}$ (eV)	−0.008	−0.094	−0.141	−0.194	−0.158	−0.146	−0.185
$\bar{E_{ads}}$ (eV/H_2_)	−0.008	−0.051	−0.081	−0.109	−0.119	−0.124	−0.132

**Table 6:** Hydrogen adsorption energy and average adsorption energy of Li-VGDY.

N (H_2_)	1	2	3	4	5	6	7
$E_{ads}$ (eV)	−0.178	−0.149	−0.120	−0.155	−0.171	−0.146	−0.162
$\bar{E_{ads}}$ (eV/H_2_)	−0.178	−0.164	−0.149	−0.151	−0.155	−0.153	−0.154
